# Significantly Improved Pharmacokinetics Enhances *In Vivo* Efficacy of APX001 against Echinocandin- and Multidrug-Resistant Candida Isolates in a Mouse Model of Invasive Candidiasis

**DOI:** 10.1128/AAC.00425-18

**Published:** 2018-09-24

**Authors:** Yanan Zhao, Min Hee Lee, Padmaja Paderu, Annie Lee, Cristina Jimenez-Ortigosa, Steven Park, Robert S. Mansbach, Karen Joy Shaw, David S. Perlin

**Affiliations:** a Public Health Research Institute, New Jersey Medical School-Rutgers Biomedical and Health Sciences, Newark, New Jersey, USA; b Amplyx Pharmaceuticals, Inc., San Diego, California, USA

**Keywords:** APX001, APX001A, pharmacokinetics, efficacy, 1-aminobenzotriazole, *Candida*

## Abstract

APX001 is a first-in-class, intravenous and orally available, broad-spectrum antifungal agent in clinical development for the treatment of life-threatening invasive fungal infections. The half-life of APX001A, the active moiety of APX001, is significantly shorter in mice than in humans (1.4 to 2.75 h in mice versus 2 to 2.5 days in humans), making the exploration of efficacy in mouse models difficult. After pretreatment with 1-aminobenzotriazole (ABT), a nonspecific cytochrome P450 inhibitor, greatly increased plasma APX001A exposure was observed in mice of different strains and of both genders. As a consequence, 26 mg/kg APX001 plus ABT sterilized kidneys in mice infected with Candida albicans, while APX001 alone at the same dose resulted in a modest burden reduction of only 0.2 log_10_ CFU/g, relative to the vehicle control. In the presence of ABT, 2 days of once-daily dosing with APX001 at 26 mg/kg also demonstrated significant *in vivo* efficacy in the treatment of Candida glabrata infections in mice. Potent kidney burden reduction was achieved in mice infected with susceptible, echinocandin-resistant, or multidrug-resistant strains. In contrast, the standard of care (micafungin) was ineffective in treating infections caused by the resistant C. glabrata isolates.

## Abstract (precis)

APX001 is a first-in-class, intravenous and orally available, broad-spectrum antifungal agent in clinical development for the treatment of life-threatening invasive fungal infections. The half-life of APX001A, the active moiety of APX001, is significantly shorter in mice than in humans (1.4 to 2.75 h in mice versus 2 to 2.5 days in humans), making the exploration of efficacy in mouse models difficult.

## INTRODUCTION

APX001, the *N*-phosphonooxymethyl prodrug of APX001A, is a first-in-class, small-molecule, investigational drug in clinical development for the treatment of life-threatening invasive fungal infections. APX001A inhibits the highly conserved fungal enzyme Gwt1, a key component of the glycosylphosphatidylinositol biosynthesis pathway, hence compromising the growth of major fungal pathogens (1, 2). Potent activity of APX001A/APX001 against a broad spectrum of Candida and Aspergillus species was demonstrated by extensive *in vitro* susceptibility assessments, as well as some *in vivo* evaluations (1–7). A long half-life of ∼2.5 days was observed in phase 1 clinical trials with APX001, in which safety, tolerability, and pharmacokinetics (PK) in healthy human subjects were assessed (8, 9). In comparison, a surprisingly short half-life, ranging from 1.40 to 2.75 h, was reported in a recent PK and pharmacodynamic (PD) study utilizing mouse models of invasive candidiasis (10). Mouse candidiasis models have historically been used for antifungal drug discovery and development, because data derived from mouse studies are deemed to provide valuable insights relevant to clinical responses. However, such a marked PK difference in half-lives between humans and mice is likely a result of drug metabolism differences in these two mammalian species. It is well known that cytochrome P450 (CYP) enzymes are a major source of variability in drug PK values and responses (11). The metabolism of drugs by CYPs commonly results in reduction in pharmacological activity, compared to the parent compound, as a consequence of both the chemical structure alterations and increased elimination (12). APX001A is biotransformed to multiple phase I and phase II metabolites, including primarily oxidized, dealkylated, and deaminated forms and secondarily conjugated metabolites. Reaction phenotyping experiments have determined that multiple CYP isozymes, including CYP2C19, CYP2D6, CYP3A4, and CYP3A5, mediate the formation of phase I metabolites in human liver microsomes. Different patterns of APX001A metabolites have been observed after incubation with mouse, rat, dog, monkey, and human cryopreserved hepatocytes. Among the species tested, mouse hepatocytes produced the greatest numbers of metabolites, followed by rat, monkey, dog, and human hepatocytes; conjugated metabolites were detected only in animal samples and not in human samples. No human-specific metabolites were observed. (R. S. Mansbach, unpublished data).

The substantially expedited clearance of APX001A in mice resulting from rapid CYP-dependent metabolism is likely to lead to reduced efficacy, thereby confounding effective preclinical evaluation of this compound. The molecule 1-aminobenzotriazole (ABT) is commonly used as a nonspecific mechanism-based inhibitor of CYPs (13). The physiochemical properties of ABT make it very helpful as a tool to increase the exposure of coadministered molecules for *in vivo* studies. The aim of this proof-of-concept study was to evaluate the PK properties and *in vivo* efficacy of APX001 with greater sustained drug exposure in mice, in the presence of ABT, against infections caused by Candida albicans and Candida glabrata, including echinocandin-resistant (ER) and multidrug-resistant (MDR) isolates.

## RESULTS

### Antifungal susceptibility testing.

The MIC values of APX001A and comparators (micafungin, fluconazole, and voriconazole) are shown in Table 1. APX001A was highly active against all Candida isolates included in this study, with MIC values comparable to or lower than those of other tested antifungals. Even for the ER and MDR C. glabrata strains, APX001A MIC values were low or were only slightly elevated (1- to 4-fold) to 0.125 μg/ml.

**TABLE 1:** *In vitro* susceptibility profiles of strains used in the efficacy study

Species and isolate	Phenotype	Pdr1	Fks1/2	MIC (μg/ml)^*a*^					
				APX001A, 24 h	MCF, 24 h	FLC		VRC	
						24 h	48 h	24 h	48 h
C. albicans									
SC5314	WT	NA^*b*^	WT	0.016	0.016	1	1	0.03	0.03
C. glabrata									
ATCC 2001	WT	WT	WT	0.03	0.03	2	4	0.03	0.03
DPL40	ER	WT	Fks1-S629P	0.03	2	8	8	0.125	0.125
DPL-d249	MDR	G1079R	Fks2-S663P	0.125	16	>64	>64	2	4

a MCF, micafungin; FLC, fluconazole; VRC, voriconazole.

b NA, not applicable.

### *In vitro* assessment of ABT activity and interaction with APX001A.

MIC assays were performed for ABT by utilizing a concentration range of 0.016 to 16 μg/ml, and no growth inhibition was observed for any of the fungal species tested, including C. albicans, Cryptococcus neoformans, Aspergillus fumigatus, and Fusarium solani. The combination of APX001A and ABT was evaluated in synergy checkerboard assays utilizing 3 species (C. albicans, C. neoformans, and A. fumigatus). No detectable *in vitro* interactions (synergistic, additive, or antagonistic effects) were observed between ABT and APX001A in growth inhibition assays.

### Pharmacokinetics.

The impact of CYPs on the drug exposure of APX001A was first investigated in a single-dose PK experiment in healthy male mice following an intravenous (i.v.) dose of APX001 at 26 mg/kg. In mice pretreated with ABT, the APX001A maximum drug concentration (*C*_max_) and area under the concentration-time curve (AUC) values were considerably higher and the elimination half-life of 6.32 h was remarkably longer than the values obtained without oral ABT pretreatment (Table 2) or observed previously (10). PK parameters were similar in the humanized PXR-CAR-CYP3A4/3A7 mice and the C56BL/6 wild-type (WT) mice without ABT treatment, indicating that CYP3A4 is not substantially involved in APX001A metabolism in mice. In a second single-dose PK study undertaken in female CD-1 mice with APX001 delivered by intraperitoneal (i.p.) injection, ABT altered the drug exposure in a similar manner. As shown in Table 2, AUC values with and without ABT pretreatment were 68.60 and 7.49 μg · h/ml, respectively, representing a 9.2-fold increase in exposure in the presence of ABT. The half-life also increased from 1.3 h to 8.8 h in the presence of ABT. Thus, the administration of ABT improved the PK of APX001A in different strains of mice, in both sexes, and with different routes of administration (i.v. and i.p.). Studies (in which drug was administered i.v., orally, i.p., or subcutaneously) that examined doses of 3.25 mg/kg to 52 mg/kg APX001 without ABT demonstrated linear PK; similarly, linearity was observed in a preliminary study of 26 mg/kg versus 52 mg/kg APX001 plus ABT (data not shown).

**TABLE 2:** Pharmacokinetic parameters for APX001A in various mouse strains following a single i.v. or i.p. dosing of 26 mg/kg APX001 alone or after oral pretreatment with 100 mg/kg ABT

Mouse strain	Treatment	*C*_max_ (μg/ml)	AUC^*a*^ (μg · h/ml)	Half-life (h)
C57BL6 WT	APX001 i.v.	3.91	5.38	ND^*b*^
C57BL6 WT	APX001 i.v. + ABT	10.10	111.0	6.32
PXR-CAR-CYP3A4/3A7	APX001 i.v.	5.09	5.20	0.88
CD-1	APX001 i.p.	4.84	7.49	1.3
CD-1	APX001 i.p. + ABT	7.29	68.60	8.8

a The time course for the i.v. experiment was predose and 0.5, 1, 2, 4, 6, 8, and 24 h postdose. The time course for the i.p. experiment was predose and 0.083, 0.25, 0.5, 1, 2, 4, 6, 8, 12, and 24 h postdose. For both experiments, AUC values are from time zero to the time of the last measured concentration.

b ND, not determined (*r*^2^ < 0.85, or insufficient concentration-time data for the terminal phase).

### *In vivo* efficacy evaluation.

**FIG 1:**
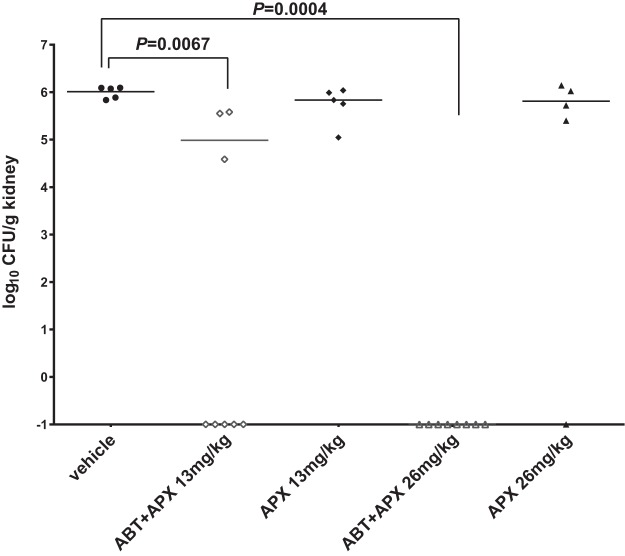
Kidney burdens in mice at 48 h after infection with C. albicans SC5314. Treatments without ABT pretreatment were administered by i.p. injection, i.e., APX001 (APX) at 13 mg/kg once daily or 26 mg/kg once daily or vehicle control once daily (*n* = 5 per group). Another 2 groups of mice (*n* = 8 per group) were given oral pretreatment with 100 mg/kg ABT 2 h prior to each APX001 dosing (at 13 mg/kg once daily or 26 mg/kg once daily). Differences in kidney burdens among groups were assessed by ANOVA with the *post hoc* Dunn's test for multiple comparisons. *P* values of <0.05 were considered statistically significant.

To evaluate whether the increased exposure of APX001A resulting from the administration of ABT translated into enhanced efficacy, we first assessed kidney fungal burdens following 2 doses of APX001, with or without ABT pretreatment, in neutropenic mice systemically challenged with C. albicans strain SC5314. At the start of therapy, mice had kidney burdens of 3.7 ± 0.2 log_10_ CFU/g. The organism grew to 2.3 ± 0.1 log_10_ CFU/g of kidney within 48 h in untreated control mice. As illustrated in Fig. 1, APX001 alone at both dosages resulted in modest burden reductions of 0.2 log_10_ CFU/g, relative to kidney burdens of 6.0 ± 0.1 log_10_ CFU/g in vehicle-treated mice. In comparison, pretreatment with ABT markedly improved the *in vivo* efficacy of APX001, with an average burden reduction of 1.1 log_10_ CFU/g being observed with the 13 mg/kg treatment and complete sterilization being achieved in all mice (*n* = 8) treated with 26 mg/kg APX001. Kidney sterilization was also achieved in 5 of 8 mice treated with 13 mg/kg APX001. Based on the PK parameters shown in Table 2 and the fact that no notable accumulation was seen with 7 daily doses of 26 mg/kg APX001, with or without ABT pretreatment (data not shown), the predicted daily APX001A AUC values for the 4 APX001 treatment groups were as follows: 13 mg/kg, 3.7 μg · h/ml; 13 mg/kg plus ABT, 34 μg · h/ml; 26 mg/kg, 7.5 μg · h/ml; 26 mg/kg plus ABT, 69 μg · h/ml.

**FIG 2:**
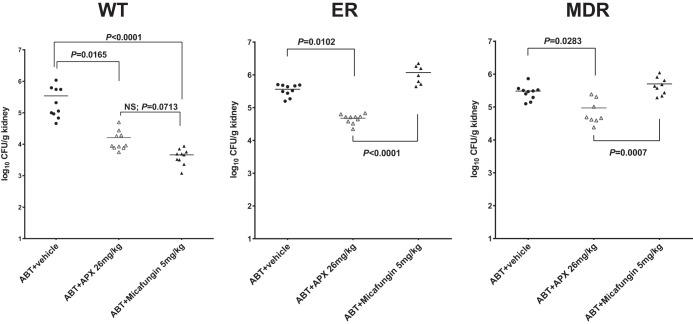
Kidney burdens in mice at 48 h after infection with C. glabrata ATCC 2001 (WT), DPL40 (ER), or DPL-d249 (MDR). Oral pretreatment with ABT was applied to all groups 2 h prior to each dosing. Once-daily i.p. treatments in each infection cohort were 26 mg/kg APX001 (APX), 5 mg/kg micafungin, or vehicle control (*n* = 10 per group). Differences in kidney burdens among groups were assessed by ANOVA with the *post hoc* Dunn's test for multiple comparisons. Dead mice (3 infected with the ER strain and treated with micafungin, 2 infected with the MDR strain and treated with APX001, and 1 infected with the MDR strain and treated with micafungin) were excluded from statistical analyses. *P* values of <0.05 were considered statistically significant.

Moving forward, the *in vivo* efficacy of APX001 in the presence of ABT was further assessed against 3 C. glabrata isolates, including susceptible strain ATCC 2001, ER strain DPL 40 (Fks1-S629P), and MDR (ER and azole-resistant) strain DPL-d249 (Pdr1-G1079R and Fks2-S663P). Micafungin at 5 mg/kg (a conventional dose, which has been shown to be effective against disseminated candidiasis in mice and results in free-drug AUC [*f*AUC] values that approximate the *f*AUC values in humans receiving 100 to 150 mg/day) was included as the comparator in this evaluation (14–19). Kidney burdens at the start of therapy were 3.9 ± 0.2 log_10_ CFU/g and increased 1.6 ± 0.2 log_10_ CFU/g in untreated control mice at the end of therapy; the growth values were similar among the 3 strains. By 48 h after challenge with the WT strain, mice had mean kidney burdens of 5.5, 4.2, and 3.7 log_10_ CFU/g as a result of receiving 2 doses of vehicle, 26 mg/kg APX001, and 5 mg/kg micafungin, respectively (Fig. 2). In this case, the efficacy of APX001 was comparable to that of micafungin (*P* = 0.0713), and both drugs led to significant burden reductions, relative to vehicle-treated controls. For mice infected with the ER strain, however, only APX001 was effective, with a burden reduction of 0.9 log_10_ CFU/g (*P* = 0.0102), in contrast to the burden increase of 0.5 log_10_ CFU/g observed with micafungin. Significant *in vivo* efficacy was also achieved with APX001 in mice infected with MDR C. glabrata, with a burden reduction of ∼0.5 log_10_ CFU/g (*P* = 0.0283), while micafungin was not effective at all. It should be noted that death was observed for mice infected with either the ER strain or the MDR strain. Specifically, 3 mice infected with the ER strain and treated with micafungin died at 24 h postinfection. Of the mice infected with the MDR strain, 2 from the APX001 group and 1 from the micafungin group died overnight before sacrifice at 48 h. The dead mice were excluded from statistical analyses.

## DISCUSSION

Despite advances in antifungal therapy, invasive fungal infections remain major causes of morbidity and death among immunocompromised patients (20). Three major classes of antifungal agents are currently available to treat invasive fungal infections; however, each class has its limitations, due to either limited spectra, toxicity, delivery issues, major drug interactions, or issues of drug resistance (21). Therefore, new antifungal agents are urgently needed. As a first-in-class agent, APX001A has demonstrated potent *in vitro* activity against a wide spectrum of fungal pathogens (1–5). *In vivo* efficacy of the prodrug APX001 was also achieved with dosing two or three times a day (3, 6, 7). However, a substantially shorter half-life in mice, requiring multiple daily doses, has limited the evaluation of the *in vivo* efficacy of APX001 using mouse models, especially when 7-day dosing is required. Herein, we demonstrated that strikingly increased exposure of APX001A in mice was obtained with administration of ABT, a nonspecific CYP inhibitor, enabling a once-daily dosing regimen as well as facilitating more efficient efficacy evaluation with a dosing interval that more closely mimics the dosing interval in human subjects.

Consistent with the markedly enhanced drug exposure resulting from the preadministration of ABT, complete elimination of kidney burdens was observed in all mice treated with 26 mg/kg APX001 and 5 of 8 mice treated with 13 mg/kg APX001 in combination with ABT against systemic C. albicans infection. In comparison, APX001 alone at both doses resulted in modest kidney burden reductions of only 0.2 log_10_ CFU/g, relative to untreated controls. Notably, this impressive tissue sterilization effect was not reported in any previous studies assessing the kidney burden reduction efficacy of APX001 in mice systemically infected with C. albicans strains, even with much higher doses and more frequent dosing (6, 7, 10). It should be noted that ABT alone did not have any *in vitro* inhibitory effects on the growth of several yeasts and molds (K. J. Shaw, unpublished data), and it did not show any *in vivo* antifungal effects with C. albicans- or C. glabrata-infected mice in our mouse models (Y. Zhao, unpublished data).

With ABT preadministration, once-daily dosing of 26 mg/kg APX001 also demonstrated efficacy in mice infected with C. glabrata strains, with kidney burden reductions of 1.3, 0.9, and 0.5 log_10_ CFU/g against susceptible, ER, and MDR strains, respectively, within the 48-h study period. Although kidney sterilization (as observed in the C. albicans experiment) was not achieved in this model, fungal burden reductions, relative to the vehicle control, were still prominent, as similar effects were obtained in previous studies only using higher and more frequent drug doses and/or longer courses of dosing to treat either C. albicans- or C. glabrata-infected mice (3, 7, 10). More importantly, the statistically significant efficacy achieved with the combination of ABT and APX001 against both ER and MDR C. glabrata strains is highly encouraging and may have clinical implications for the treatment of resistant C. glabrata infections, because the standard of care (micafungin) is ineffective in such cases due to well-known resistance mechanisms.

There are some limitations in this study, including only 1 C. albicans strain being used for *in vivo* efficacy evaluation and only 1 dose level (26 mg/kg APX001 daily plus ABT) and 1 treatment endpoint (48 h) being used to evaluate efficacy against C. glabrata infections. We acknowledge that higher doses and/or a longer course of therapy might result in increased drug exposure and enhanced efficacy. In this limited proof-of-concept study, however, we have demonstrated the utility of ABT to facilitate efficient efficacy evaluations in mice. More extensive studies are needed to explore the optimal dosing strategy for the combination of ABT and APX001. Some individual variability was noted in mice infected with C. albicans and treated with 13 mg/kg APX001 plus ABT. The underlying mechanism of such dichotomous burden distribution remains unknown and warrants further investigation. We also note that a few early deaths occurred in the C. glabrata efficacy experiment, which may reflect a potential drug interaction between ABT and cyclophosphamide, which is used for immunosuppression of the mice.

In summary, the administration of ABT significantly increased APX001A exposure in mice, thereby facilitating more efficient evaluation of APX001 efficacy using mouse models. Using this strategy, we demonstrated that APX001 shows promise as an effective antifungal agent for the treatment of susceptible, ER, or MDR Candida infections.

## MATERIALS AND METHODS

### Fungal strains, antifungal drugs, and ABT.

C. albicans strain SC5314 and C. glabrata strains ATCC 2001, DPL40, and DPL-d249 were grown on yeast extract-peptone-dextrose (YPD) agar plates. All 4 Candida strains were used for both *in vitro* susceptibility testing and *in vivo* efficacy evaluation. C. albicans strain 90028, C. neoformans strain H99, A. fumigatus strain MYA3626, and F. solani strain MYA3636 were used for the ABT susceptibility testing and checkerboard assays. APX001A, APX001 (Amplyx Pharmaceuticals Inc., San Diego, CA, USA), and micafungin (Astellas Pharma Inc., Tokyo, Japan) were obtained as standard powders from their manufacturers. Antifungal drug formulation was performed according to the manufacturer's instructions. ABT was purchased from Sigma and was dissolved in sterile water at 20 mg/ml prior to use.

### *In vitro* susceptibility testing and synergy assay.

The MICs of APX001A and comparator antifungal drugs (micafungin, fluconazole, and voriconazole) were determined for all 4 strains with the broth microdilution method, following Clinical and Laboratory Standards Institute (CLSI) guidelines (22). All MIC assays were performed in duplicate and repeated three times. Candida parapsilosis ATCC 22019 and Candida krusei ATCC 6258 were used as quality control strains for all susceptibility testing. MICs were read at 24 h and/or 48 h and were defined as the lowest concentrations to inhibit growth by ≥50%, compared to the growth control. The checkerboard microdilution assay was performed as described previously, to evaluate the interaction of ABT and APX001A and to determine whether the compounds displayed synergy, additivity, or antagonism (23–27).

### Animals.

Four-week-old male humanized PXR-CAR-CYP3A4/3A7 mice (Taconic, Rensselaer, NY), male humanized PXR-CAR-CYP3A4/3A7 mice (Taconic), and 6-week-old female CD-1 mice were used in the PK studies. Female 6-week-old BALB/c mice (Charles River Laboratories) were used in the efficacy studies. All mice were housed in the animal biosafety level 2 research animal facility of the Public Health Research Institute, a center of the New Jersey Medical School, Rutgers University. Our animal facility follows the Public Health Service and National Institutes of Health policies for humane care and use of laboratory animals. All experimental protocols were approved by the Rutgers University Institutional Animal Care and Use Committee.

### PK studies.

Two sets of single-dose PK studies were performed. In the first set, 4-week-old male C56BL/6 WT mice were randomized into 2 groups (*n* = 22 per group), receiving APX001 treatment alone or after pretreatment with a single oral dose of ABT at 100 mg/kg. ABT was administered 2 h prior to APX001 dosing. A group of male humanized PXR-CAR-CYP3A4/3A7 mice (Taconic, Rensselaer, NY) (*n* = 22) was also included in the experiment, receiving APX001 treatment only. Blood was collected before the dose (*n* = 1) and 0.5, 1, 2, 4, 6, 8, and 24 h after the dose (*n* = 3 per group per time point). Plasma was prepared within 1 h after collection and was stored frozen at −20°C until analysis. APX001A (active metabolite) concentrations in plasma were measured using liquid chromatography-tandem mass spectrometry (LC-MS/MS), with a lower limit of quantitation of 50.0 ng/ml. Noncompartmental PK analysis was performed at QPS with sparse sampling plasma concentration-time data, using the extravascular model in Phoenix WinNonlin v6.3 (Pharsight Corp., Princeton, NJ) for APX001A, and *C*_max_, AUC_last_, and half-life values were calculated. The second set of PK experiments was performed using female CD-1 mice. Mice were administered a single dose of 26 mg/kg APX001 by i.p. injection, with or without pretreatment with 100 mg/kg ABT. Plasma was collected before the dose (*n* = 1) and 0.083, 0.25, 0.5, 1, 2, 4, 6, 8, 12, and 24 h after the dose (*n* = 3 per group per time point). Measurements of APX001A concentrations in plasma and calculation of PK parameters were as described above.

### *In vivo* efficacy evaluation in mice with invasive candidiasis.

A well-established neutropenic mouse model of disseminated candidiasis was used for the efficacy study (28). Mice were rendered neutropenic through administration of cyclophosphamide at 150 mg/kg on day −4 and 100 mg/kg on day −1. The organisms were subcultured overnight in liquid YPD medium at 37°C, with shaking. Cells were collected by centrifugation, washed twice with sterile phosphate-buffered saline (PBS), and counted with a hemocytometer. The inoculum was adjusted to the desired concentration, and 50 μl was used to infect each mouse. The actual infection dose was verified by assessment of viable counts on YPD plates spread with proper dilutions of the inoculum and incubated at 37°C for 24 h. To assess the activity of APX001 against infections caused by C. albicans, mice were infected on day 0 with 5 × 10^4^ CFU of C. albicans SC5314, via retroorbital injection. Groups of 5 or 8 mice (*n* = 5 for groups without ABT treatment and *n* = 8 for groups with ABT pretreatment) were randomly assigned to 5 treatment arms, i.e., APX001 at 13 or 26 mg/kg, alone or with ABT, and vehicle control. APX001 and vehicle treatments were administered i.p. at 1 and 24 h postinfection. ABT was administered at 100 mg/kg, via oral gavage, 2 h prior to each APX001 dose for the APX001 plus ABT group. Mice were sacrificed at 48 h postinfection, and kidney burdens were measured by quantitative culture. In the experiment to evaluate the efficacy for treating infections caused by C. glabrata, mice underwent the induction of neutropenia as described above and were infected i.v. with ∼2.5 × 10^6^ CFU of C. glabrata ATCC 2001, DPL40, or DPL-d249. ABT pretreatment was performed for all mice at 2 h prior to each dosing. Groups of 10 mice were treated with APX001 at 26 mg/kg, micafungin at 5 mg/kg, or vehicle in the same manner as in the C. albicans infection model. All mice were sacrificed 48 h postinfection, and kidney burdens were evaluated and compared. To assess burdens at the start of therapy, 5 extra mice infected with the same inoculum were included in both experiments for each infecting strain. All graphical data are expressed as grouped data points with means and were statistically analyzed by analysis of variance (ANOVA) using Prism 6 software (GraphPad Software, Inc., San Diego, CA). Burden differences between testing and control groups were assessed by *post hoc* analysis, using Dunn's multiple-comparison test. *P* values of <0.05 were considered statistically significant.
